# Genome-Wide Characterization of the ZIP Transporter Family in Sea Island Cotton (*Gossypium barbadense* L.) and Expression Profiling Under Heavy Metal and Pathogen Stresses

**DOI:** 10.3390/biology15171455

**Published:** 2026-08-26

**Authors:** Yahui Deng, Nan Zhao, Jidi Sun, Jianping Li, Meng Wang, Yifan Wang, Zhiqing Liu, Zixin Zhou, Caixia Li, Lingfang Ran, Yaohua Li, Jing Yang, Jiahui Zhu, Alifu Aierxi, Wumaierjiang Kuerban, Jie Kong, Weiran Wang

**Affiliations:** 1 Xinjiang Key Laboratory of Cotton Genetic Improvement and Intelligent Production, National Cotton Engineering Technology Research Center, Cotton Research Institute of Xinjiang Uyghur Autonomous Region Academy of Agricultural Sciences, Urumqi 830091, China; huiyad0330@163.com (Y.D.); nan_zhao@cau.edu.cn (N.Z.);; 2 College of Agronomy, Xinjiang Agricultural University, 311 Nongda East Road, Urumqi 830052, China

**Keywords:** *Gossypium barbadense*, ZIP family, heavy metal transport, Verticillium wilt, cadmium stress, fiber development

## Abstract

*G. barbadense* represents an indispensable germplasm resource for high-quality textile fiber and disease resistance; nevertheless, systematic information regarding its ZRT/IRT-like protein (ZIP) gene family remains limited. Here, a total of 46 GbZIP genes were identified across the *G. barbadense* genome. Comprehensive bioinformatic investigations revealed uneven chromosomal distribution and confirmed that segmental/whole-genome duplications, supplemented by localized tandem duplications, drove family expansion. Members clustered within the same phylogenetic clades shared conserved motif organization and gene architecture, while promoter regions harbored abundant cis-acting elements associated with phytohormone and stress signaling. Transcriptome profiling indicated distinct expression patterns across vegetative/reproductive tissues, fiber and ovule developmental stages, and diverse abiotic stress conditions (cold, hot, drought, and salt). Quantitative Real-Time PCR (qRT-PCR) further validated that several GbZIP candidates exhibited temporal expression variations upon exposure to cadmium toxicity, *V. dahliae* infection, and combined Cd-*V. dahliae* stress. Specifically, *GbZIP13*, *GbZIP18*, *GbZIP27*, and *GbZIP36* displayed prominent broad-spectrum responses to all three stress conditions, whereas *GbZIP16*, *GbZIP29*, and *GbZIP30* showed stress-specific regulatory divergence. Overall, this study aims to systematically analyze the evolutionary characteristics and expression patterns of the GbZIP family, and to specifically evaluate the response differences under Cd stress, *V. dahliae* stress, and combined stress, in order to identify potential key candidate genes.

## Simple Summary

Sea Island cotton (*Gossypium barbadense* L.) is widely cultivated for its high-grade extra-long staple fiber and excellent disease resistance. However, its cultivation is increasingly threatened by soil heavy metal contamination (such as cadmium toxicity) and fungal diseases (such as Verticillium wilt caused by *Verticillium dahliae*). Zinc/iron-regulated transporter-like proteins (ZIPs) serve as essential membrane transporters that manage transition metal homeostasis and modulate stress responses in plants. In this study, we systematically identified 46 GbZIP genes across the *G. barbadense* genome and comprehensively evaluated their structural characteristics, evolutionary origin, cis-regulatory elements, and expression patterns. Transcriptional analysis demonstrated that several key GbZIP members dynamically respond to cadmium exposure, fungal infection, and their combined occurrence. These findings highlight crucial candidate genes for molecular breeding aimed at improving stress resilience and metal tolerance in premium cotton germplasm.

## 1. Introduction

Cotton (*Gossypium* spp.) stands as an indispensable economic crop globally, supplying essential natural raw materials to the textile industry. Among the four cultivated cotton species, Sea Island cotton (*Gossypium barbadense* L.) is highly valued for its superior fiber fineness, length, and strength [1]. Despite its immense agricultural value, production is severely threatened by soil-borne vascular diseases, most notably Verticillium wilt caused by the fungal pathogen *Verticillium dahliae* Kleb [2]. Following infection through the root system, *V. dahliae* colonizes xylem vessels, producing hyphae and conidia that physically obstruct water transport alongside cell-wall-degrading enzymes and phytotoxins [2]. This vascular disruption leads to severe leaf chlorosis, plant wilting, and yield declines exceeding 50% [2]. In contrast to *G. hirsutum*, which accounts for the vast majority of commercial acreage but exhibits limited genetic resistance to *V. dahliae*, *G. barbadense* germplasm harbors rich innate resistance reservoirs [3]. Genomic analyses and functional gene discoveries have pinpointed key factors governing Verticillium wilt resistance in *G. barbadense*, including *GbOSM1* [4], *GbVIP1* [5], and *GbCYP72A2* [6]. Nevertheless, functional characterization of broader resistance-related gene networks in *G. barbadense* remains incomplete.

Concurrently, modern agricultural ecosystems suffer from abiotic stress factors, including heavy metal accumulation driven by rapid industrial activities [7]. Cadmium (Cd) represents one of the most hazardous phytotoxic heavy metals, lacking known biological functions while inhibiting seed germination, biomass accumulation, and fiber elongation [7,8]. Physiological and metabolic responses to Cd vary substantially among cotton cultivars, negatively altering root ultrastructure, photosynthetic capacity, and fiber quality parameters [8]. Importantly, heavy metal contamination and fungal pathogens often coexist in field environments [7,9]. Cd accumulation disrupts cellular homeostasis and alters rhizosphere conditions, which can in turn modulate pathogen colonization and host defense signaling [7,9]. Understanding how cotton integrates responses to combined heavy metal toxicity and biotic stress represents an urgent priority for crop stress biology.

The ZRT/IRT-like protein (ZIP) superfamily constitutes a vital class of transmembrane transporters that regulate divalent metal ion uptake, intracellular trafficking, and systemic homeostasis [10]. Members of this family mediate the transport of essential micronutrients such as Zn^2+^, Fe^2+^, and Mn^2+^, while also facilitating the translocation of non-essential toxic ions such as Cd^2+^ [10,11]. Extensive studies across major crops have demonstrated the dual functions of ZIP transporters in nutrient acquisition and metal detoxification [11]. For instance, *OsZIP2* mediates root-to-shoot Cd translocation [12], rice *OsZIP1* functions as a metal efflux transporter [13], and *OsZIP3* participates in intervascular transfer alongside co-expression networks [14]. Similarly, wheat *TpZIP3-2A* [15], maize *ZmHMA3* [16], and potato *StZIP5* [17] contribute significantly to metal ion redistribution under Cd toxicity.

Despite these insights in model plants and cereals, comprehensive genome-wide analysis of the ZIP family in *G. barbadense* has not been systematically reported. Although 45 GhZIP genes were identified in *G. hirsutum*, their functional roles under combined biotic and abiotic stresses remain largely unexplored [18]. Island cotton possesses both excellent fiber quality and strong adaptability. However, in actual production, it still simultaneously faces the dual stress of heavy metal pollution and pathogen infection. Therefore, analyzing the role of the GbZIP family in multiple stresses holds significant theoretical and practical value.

In this study, GbZIP members were systematically identified from the *G. barbadense* genome. We performed comprehensive structural, phylogenetic, promoter, and synteny analyses, complemented by expression profiling across tissues, developmental stages, and abiotic stress treatments. Furthermore, protein interaction networks were modeled, and qRT-PCR was conducted to validate candidate gene expression under single and combined Cd and *V. dahliae* stresses. The findings provide valuable genetic targets and theoretical insights into the stress-adaptation networks of extra-long staple cotton.

## 2. Materials and Methods

### 2.1. Identification of GbZIP Gene Family Members

The genome sequence, protein sequences, and structural annotation files (FASTA and GFF3 formats) of *G. barbadense* acc. 3-79 (HAU 2.0) were retrieved from the CottonGen database (https://www.cottongen.org, accessed on 6 May 2026). *Arabidopsis* (*Arabidopsis thaliana*) ZIP family protein sequences were collected from TAIR (https://www.arabidopsis.org, accessed on 6 May 2026). ZIP protein sequences for *G. hirsutum* acc. TM-1 (ZJU) were also extracted from CottonGen (https://www.cottongen.org, accessed on 6 May 2026).

Candidate GbZIP members were identified using a dual search approach combining BLASTP + 2.14.0 and hidden Markov model (HMM) profile screening. Known *Arabidopsis* ZIP protein sequences were used as queries to perform BLASTP searches against the *G. barbadense* protein database (E-value < 1 × 10^−5^)). Simultaneously, the HMM profile corresponding to the ZIP domain (PF02535) was obtained from Pfam (https://www.ebi.ac.uk/interpro/entry/pfam/, accessed on 6 May 2026) and searched against the *G. barbadense* proteome using HMMER 3.4 (E-value < 1 × 10^−5^). Non-redundant hits were verified for conserved ZIP domains using InterPro (https://www.ebi.ac.uk/interpro/, accessed on 6 May 2026), the NCBI Conserved Domain Database (CDD, https://www.ncbi.nlm.nih.gov/cdd/, accessed on 6 May 2026), and SMART (http://smart.embl-heidelberg.de/, accessed on 6 May 2026) [19].

### 2.2. Physicochemical Properties and Subcellular Localization Prediction

Physicochemical parameters of GbZIP proteins, including amino acid length, theoretical isoelectric point (pI), molecular weight (MW), instability index, aliphatic index, and grand average of hydropathicity (GRAVY), were calculated using ExPASy ProtParam (https://web.expasy.org/protparam/, accessed on 10 May 2026). Subcellular localization was predicted using Plant-mPLoc (http://www.csbio.sjtu.edu.cn/bioinf/plant-multi/, accessed on 10 May 2026).

### 2.3. Chromosomal Mapping

Physical locations (chromosome assignment and coordinate boundaries) of GbZIP genes were extracted from the genome annotation files. Mapping visualizations were generated using TBtools v2.360 and refined with Adobe Illustrator CS6 [20]. Confirmed members were designated as GbZIP1 through GbZIP46 based on their physical positions on the chromosomes.

### 2.4. Phylogenetic Analysis

Multiple sequence alignments of ZIP proteins from *G. barbadense*, *G. hirsutum*, and *A. thaliana* were performed using ClustalW (gap opening penalty = 10.0, gap extension penalty = 0.2, BLOSUM weight matrix). Alignments were trimmed using Gblocks v0.91b to eliminate divergent and poorly aligned regions. whereas combined maximum likelihood (ML) trees for *G. barbadense*, *G. hirsutum*, and *A. thaliana* were constructed with 1000 bootstrap replicates. Final tree topologies were displayed using iTOL v6.7.6 [21].

### 2.5. Conserved Motif and Gene Structure Analysis

Conserved protein motifs were identified using the MEME Suite (https://meme-suite.org/meme/, accessed on 13 May 2026) with parameters set to a maximum of 10 motifs, motif width between 5 and 50 amino acids, and zero or one occurrence per sequence (zoops). Exon-intron structures were extracted from GFF3 annotations. Integrated diagrams of phylogenetic trees, conserved motifs, and gene structures were constructed using TBtools v1.098 [22].

### 2.6. Promoter Cis-Acting Element Identification

Genomic sequences 2000 bp upstream of the start codon (ATG) of each GbZIP gene were extracted and submitted to PlantCARE (http://bioinformatics.psb.ugent.be/webtools/plantcare/html/, accessed on 13 May 2026) for cis-element prediction [23]. Cis-acting elements involved in light responsiveness, hormone signaling (ABA, MeJA, SA, GA, and IAA), stress adaptation, and tissue-specific expression were categorized and visualized via heatmaps.

### 2.7. Syntenic and Gene Duplication Analysis

All-against-all BLASTP alignments (E-value < 1 × 10^−5^, top 5 matches) were conducted across the *G. barbadense* proteome. Synteny blocks and duplicated gene pairs were identified using MCScanX (https://github.com/wyp1125/MCScanX, accessed on 13 May 2026) under default parameters [24]. Duplication events were classified using the duplicate_gene_classifier module of MCScanX. Intraspecific synteny maps were visualized using the Advanced Circos tool in TBtools v2.360 [25]. The types of repetitive events, including large-scale duplication and tandem duplication, were determined based on the correlation between them and the relative positions of the genes on the chromosome.

### 2.8. Protein Tertiary Structure and Interaction Network Prediction

Transmembrane helices were predicted using TMHMM 2.0 (https://services.healthtech.dtu.dk/services/TMHMM-2.0/, accessed on 15 May 2026). Protein tertiary structure homology modeling was executed via SWISS-MODEL (https://swissmodel.expasy.org/, accessed on 15 May 2026). Protein-protein interaction (PPI) networks were constructed based on orthology mapping to *A. thaliana*. Reciprocal best hits from BLASTP searches (E-value < 1 × 10^−5^) clustering within corresponding phylogenetic clades were assigned as putative orthologs. Corresponding *Arabidopsis* IDs were queried against STRING v12.0 (https://string-db.org/, accessed on 15 May 2026) with a minimum confidence score threshold of 0.400. Interaction networks were visualized in Cytoscape v3.10.3, and topological metrics (node degree, betweenness centrality) were determined via NetworkAnalyzer [26].

### 2.9. Transcriptomic Expression Profiling

Publicly available RNA-seq datasets for *G. barbadense* acc. Hai7124 (NCBI accession SRP166405, https://www.ncbi.nlm.nih.gov/sra/?term=SRP166405, accessed on 10 April 2026) were utilized to evaluate expression across ten tissues (root, stem, leaf, petal, anther, bract, filament, pistil, sepal, and torus), ovule and fiber developmental stages, and abiotic stress conditions (cold, drought, hot, and salt). Clean reads were processed using fastp, aligned to the reference genome using HISAT2 v2.2.1 (https://daehwankimlab.github.io/hisat2/, accessed on 15 May 2026), and quantified via feature Counts (https://subread.sourceforge.net/, accessed on 15 May 2026) [27]. Read counts were converted to transcripts per million (TPM) and normalized as log_2_(TPM + 1). Expression heatmaps with row Z-score scaling were generated using TBtools [28].

### 2.10. Plant Growth, Stress Treatments, and qRT-PCR Validation

H7124 seeds with full grains were selected and sown in vermiculite. When the seedlings had developed two cotyledons, they were transferred to hydroponic bottles, and the nutrient solution (distilled water supplemented with Hoagland’s solution) was renewed every 3 days. The growth conditions were set at 28 °C/25 °C (day/night) with a 16 h/8 h light/dark photoperiod. At the three-true-leaf stage, plants were subjected to: (1) *V. dahliae* spore suspension inoculation (1 × 10^8^ spores/mL), in this study, the highly virulent *V. dahliae* strain K11 was used for inoculation, and no wounding treatment was applied; plants were simply placed in the bacterial suspension for treatment; (2) Cd stress (350 µmol/L CdCl_2_); (3) combined Cd + *V. dahliae* stress treatment was performed by simultaneous application of both stresses.; and (4) sterile water mock control [29]. Root tissues were harvested at 0, 24, 36, and 48 h post-treatment, immediately frozen in liquid nitrogen, and stored at −80 °C (three independent biological replicates).

Total RNA was extracted using the RNAprep Pure Kit (Tiangen Biotech, Beijing, China), and first-strand cDNA was synthesized using the EasyScript One-Step gDNA Removal and cDNA Synthesis SuperMix (TransGen Biotech, Beijing, China). Gene-specific primers were designed using Primer Premier 5 (Table S1). qRT-PCR was conducted on an Applied Biosystems StepOne Real-Time PCR System using TransStart Top Green qPCR SuperMix [30]. The amplification profile consisted of 95 °C for 30 s, followed by 40 cycles of 95 °C for 5 s and 60 °C for 30 s, ending with melting curve analysis. Given that both H7124 and 3-79 are well-known resistant materials against cotton wilt disease in islands, in this study, the cDNA of H7124 was used as the template for fluorescence quantitative PCR. *GbUBQ7* served as the internal reference gene, and relative expression levels were calculated using the 2^−ΔΔCt^ method. Asterisks indicate significant differences compared to 0 h (* *p* < 0.05, ** *p* < 0.01; *ns*, not significant). Three technical replicates were performed per biological sample.

## 3. Results

### 3.1. Characterization and Subcellular Localization of GbZIP Proteins

A total of 46 GbZIP family members were identified in *G. barbadense* (Table 1). The length of GbZIP proteins ranged from 221 amino acids (*GbZIP31*) to 595 amino acids (*GbZIP4*, *GbZIP24*, and *GbZIP36*), with corresponding molecular weights between 24,589.19 Da and 62,108.04 Da. Theoretical pI values varied from 5.62 to 9.25, indicating both acidic and basic proteins. Forty GbZIP members exhibited instability indices below 40, suggesting overall structural stability. Aliphatic index values ranged from 86.34 to 115.98. GRAVY values were predominantly positive for 44 members (−0.092 to 0.790), consistent with hydrophobic transmembrane properties. Subcellular localization predictions indicated that 38 GbZIP proteins localize to the plasma membrane, whereas 8 members target the chloroplast (Table 1).

### 3.2. Chromosomal Distribution

**Figure 1:**
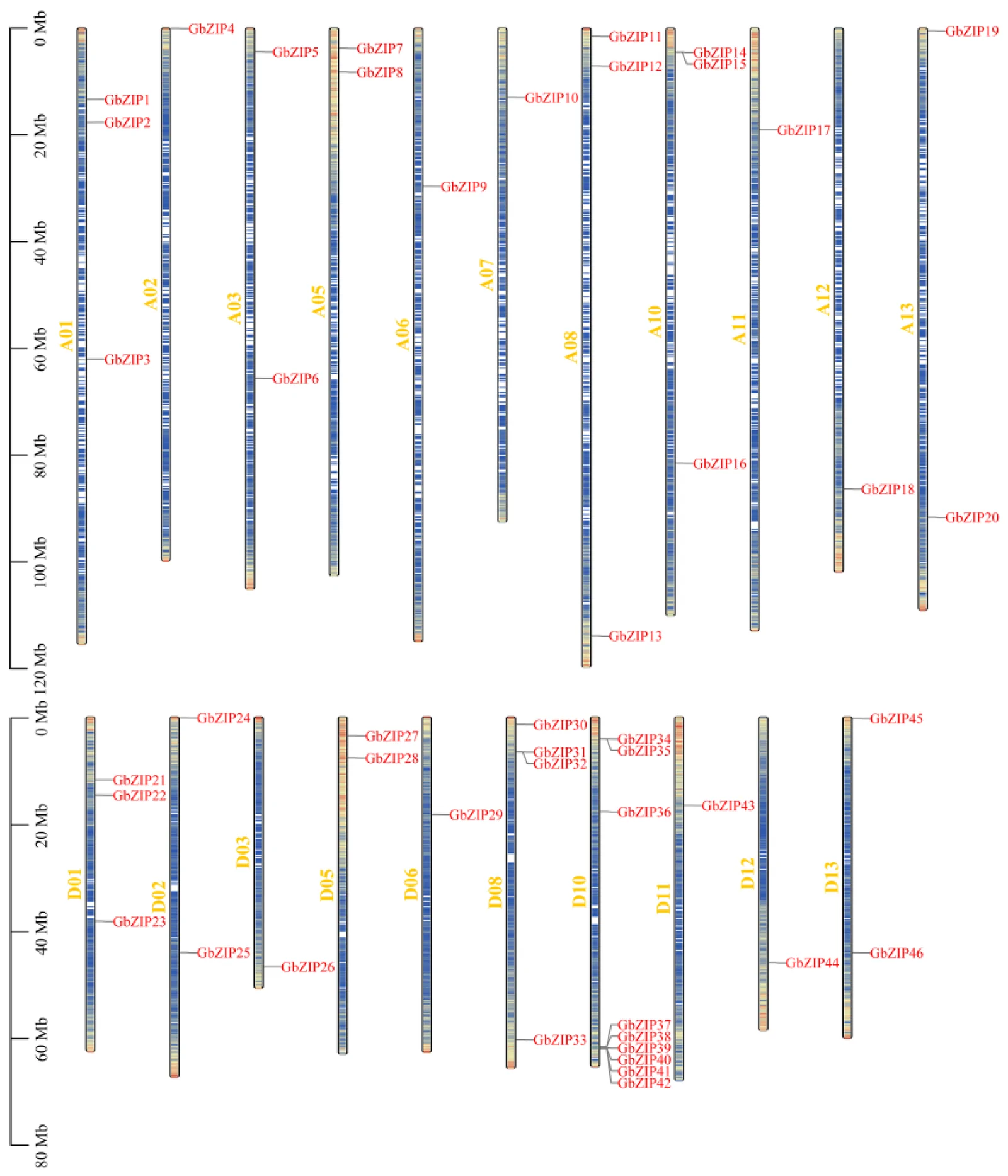
Chromosomal positioning of GbZIP genes across the *G. barbadense* subgenomes. The red font in the figure indicates the location names of the GbZIP family genes, while the yellow font represents the chromosome numbers. The blue and orange bands on the chromosome represent different chromosome segments or characteristic distributions.

Chromosomal mapping showed that 46 *GbZIP* genes were non-uniformly distributed across 21 chromosomes, with 20 assigned to the At subgenome and 26 to the Dt subgenome (Figure 1). Chromosomes A04, A09, D04, D07, and D09 contained no *GbZIP* genes. Member counts per chromosome ranged from 1 to 9, with chromosome D10 carrying the highest gene density (9 members). Localized tandem clusters were observed, such as *GbZIP7/GbZIP8* on A05 and *GbZIP37*–*GbZIP42* on D10 (Figure 1).

### 3.3. Phylogenetic Classification, Gene Structure and Conserved Motif Analyses

**Figure 2:**
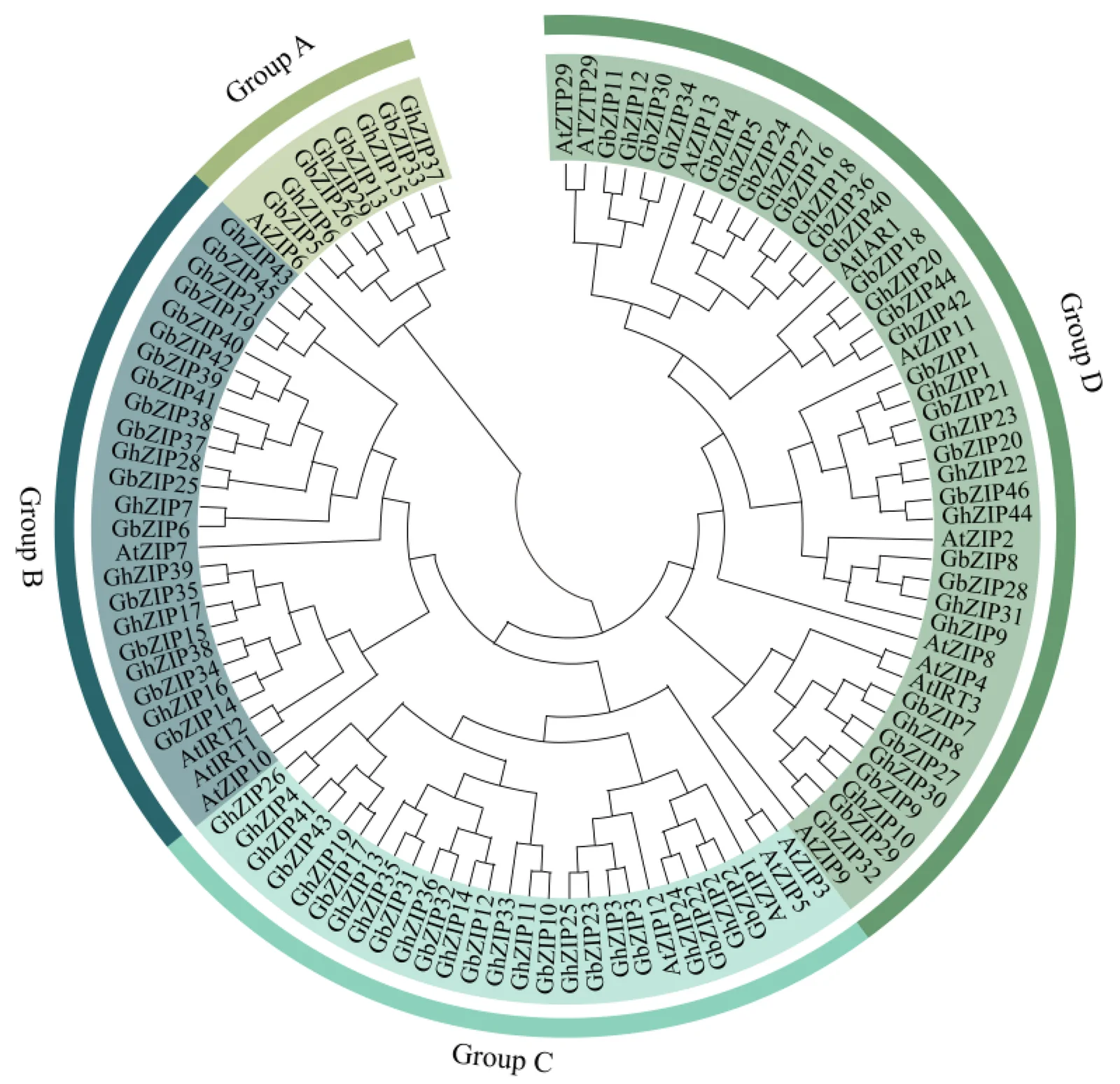
Maximum likelihood phylogenetic tree depicting evolutionary relationships among ZIP proteins from *G. barbadense*, *G. hirsutum*, and *A. thaliana*.

**Figure 3:**
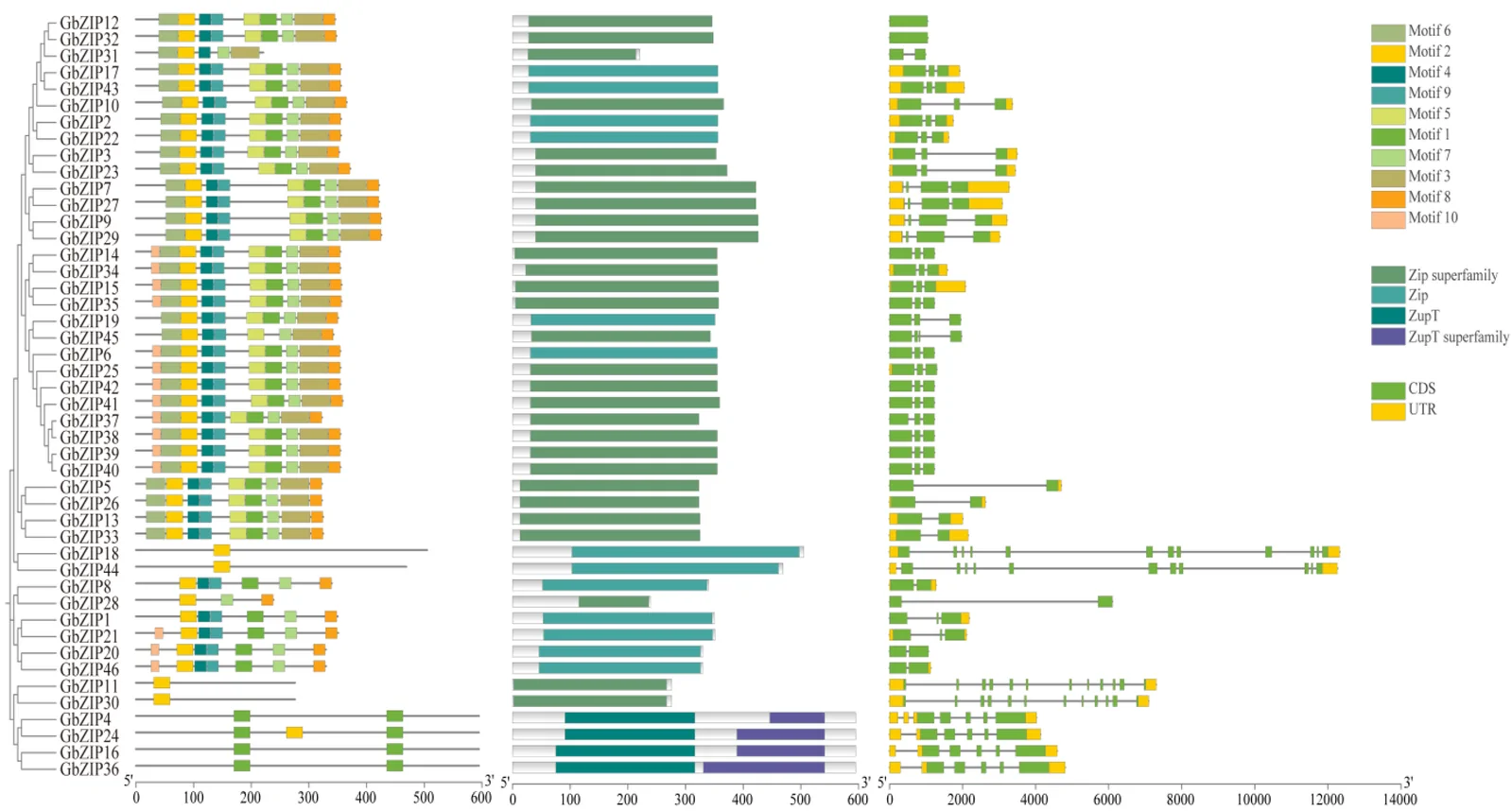
Integrated phylogenetic relationship, conserved protein motifs, domain structure, and exon-intron organizations of GbZIP family members.

Phylogenetic evaluation of ZIP proteins from *G. barbadense*, *G. hirsutum*, and *A. thaliana* grouped the members into four distinct evolutionary clades: Group A, Group B, Group C, and Group D (Figure 2). GbZIP proteins exhibited stronger orthologous clustering with *G. hirsutum* GhZIPs than with *Arabidopsis* AtZIPs, reflecting shared allotetraploid evolutionary history. Structural characterization revealed high structural conservation within phylogenetic subclades (Figure 3). Most GbZIP members possessed conserved N-terminal motif configurations and domain architectures assigned to the Zip or Zip superfamily domains. Exon-intron structural organizations were largely conserved among closely related paralogs, whereas divergent members displayed expanded intron lengths and altered motif distributions (Figure 3).

### 3.4. Syntenic Duplication and Evolutionary Relationships

**Figure 4:**
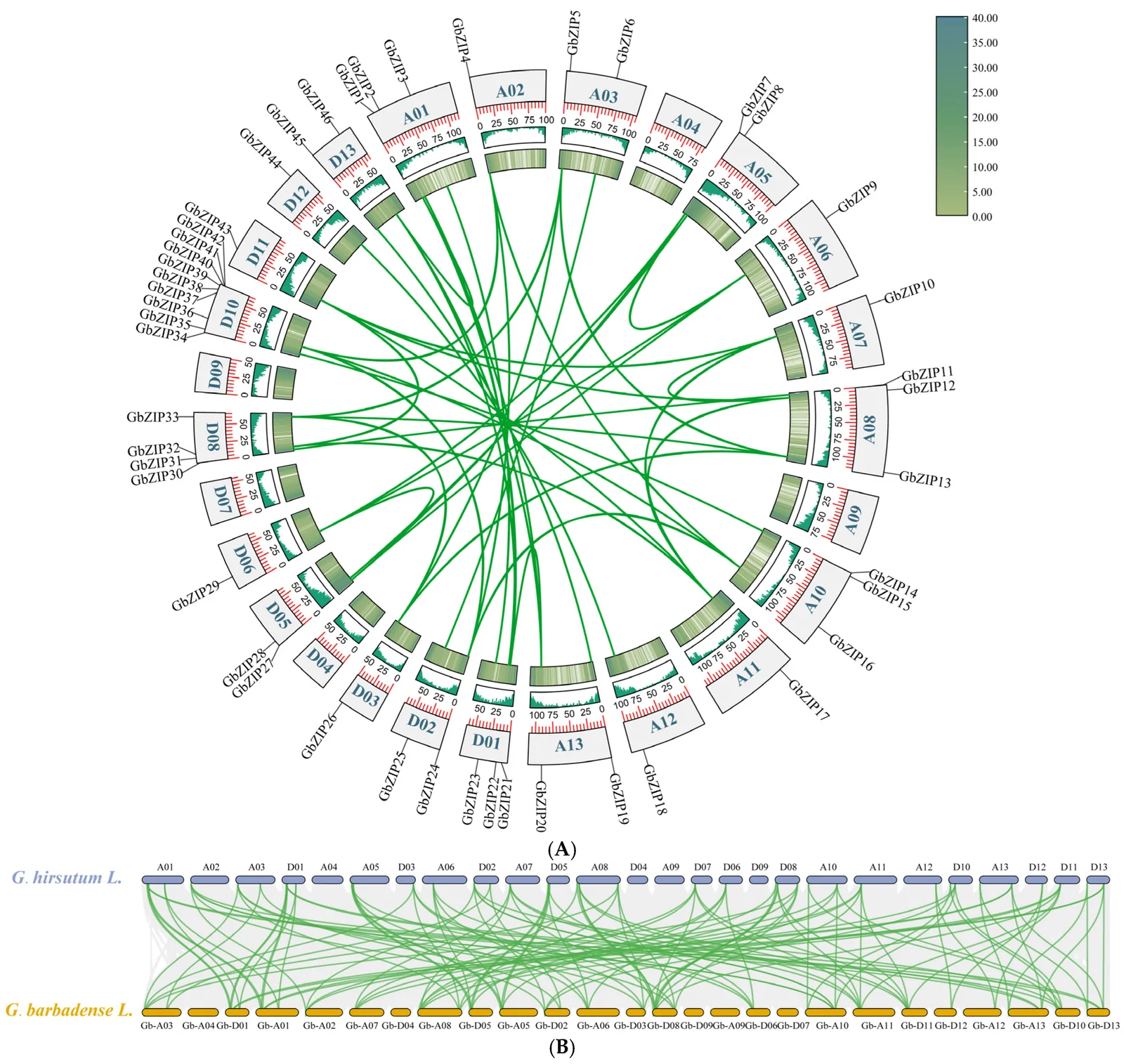
Syntenic analysis of ZIP genes. (**A**) Intraspecific collinearity among *GbZIP* genes in *G. barbadense*; (**B**) interspecific synteny between *G. barbadense* and *G. hirsutum ZIP* loci.

Intraspecific synteny analysis detected extensive collinear gene pairs across the *G. barbadense* genome, indicating that whole-genome duplication (WGD) and segmental duplication events drove the expansion of the GbZIP family (Figure 4A). Comparative interspecific synteny demonstrated high collinearity between *G. barbadense* and *G. hirsutum ZIP* loci, supporting evolutionary stability following polyploidization (Figure 4B).

### 3.5. Promoter Cis-Acting Element Profiling

**Figure 5:**
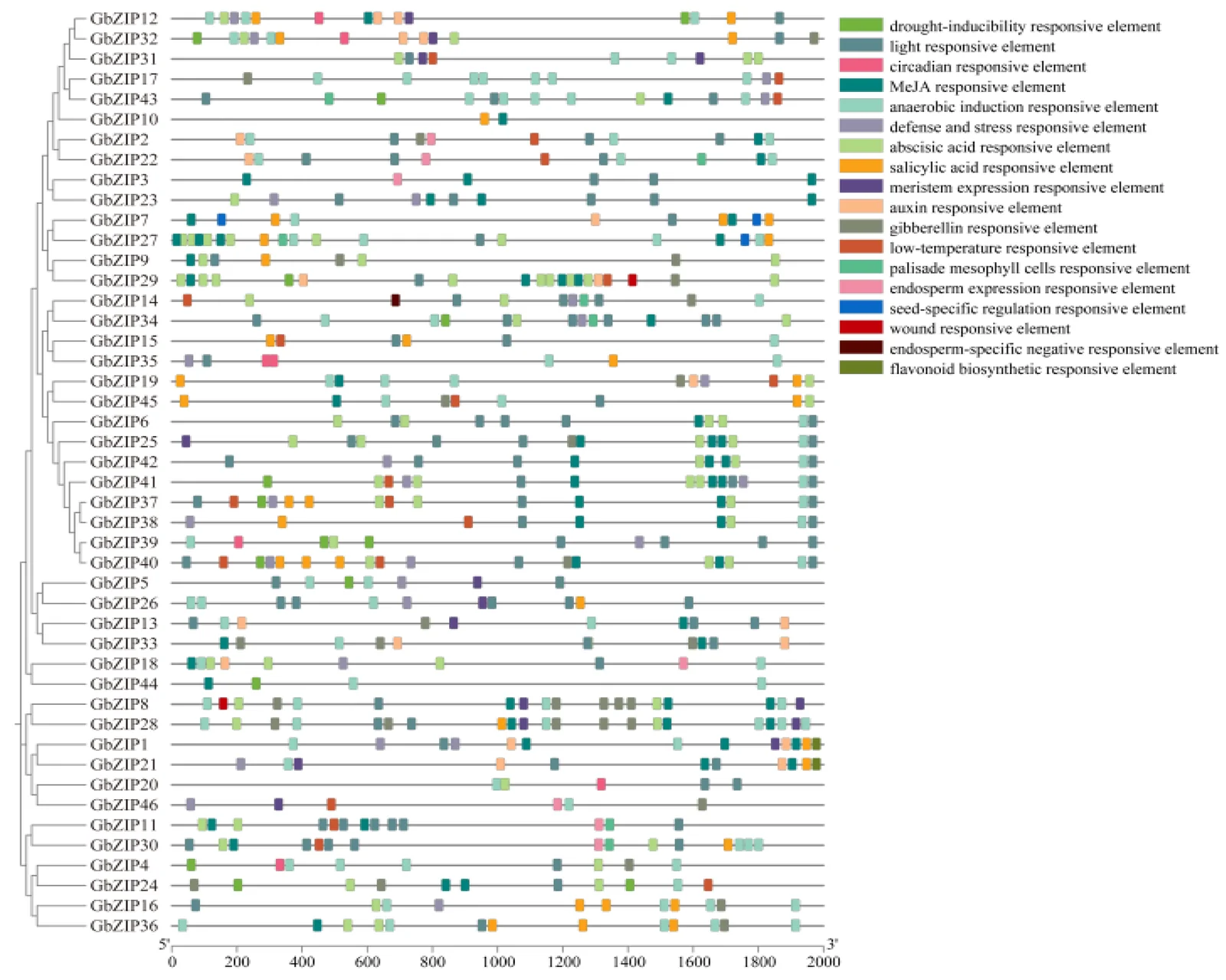
Distribution map of predicted cis-acting regulatory elements in the 2000 bp upstream promoter regions of GbZIP genes. The left panel shows the phylogenetic relationship of GbZIP genes, with the *x*-axis indicating the position in the promoter region (5′–3′) and different colored boxes representing various types of *Cis-acting* elements.

Promoter region analysis revealed widespread presence of light-responsive, phytohormone-responsive (ABA, MeJA, SA, GA, IAA), and stress-responsive elements (drought, low temperature, defense/stress, and anaerobic induction) across *GbZIP* upstream regions (Figure 5). In addition, developmental regulation elements (meristem expression, seed-specific control, endosperm expression) were identified, suggesting multi-layered transcriptional regulation under diverse physiological conditions (Figure 5).

### 3.6. Protein 3D Structure Modeling and Interaction Network

**Figure 6:**
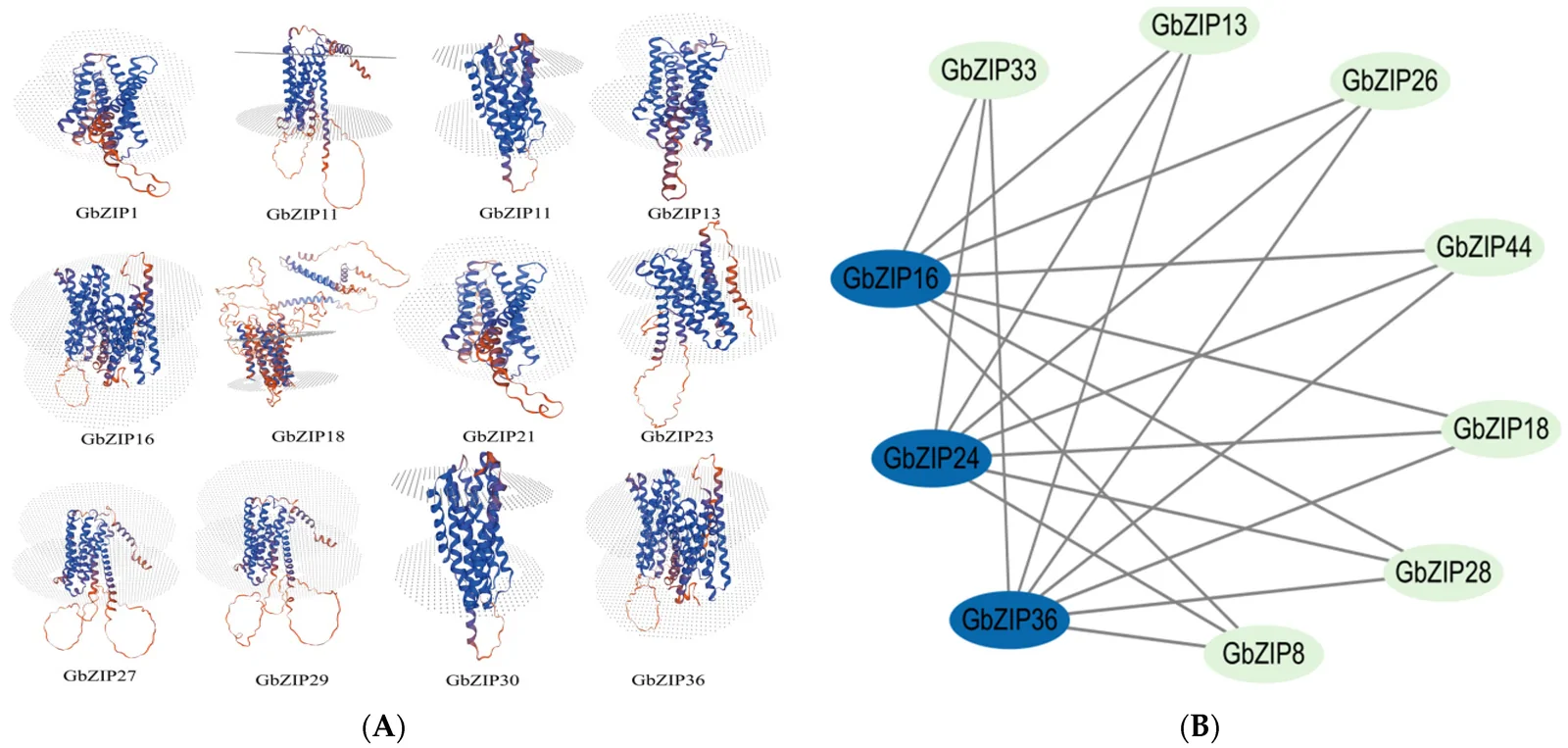
Protein 3D structure and interaction characterization. (**A**) Predicted three-dimensional tertiary structures of representative GbZIP proteins; (**B**) modeled protein–protein interaction network of the GbZIP family.

Three-dimensional structure predictions confirmed that GbZIP proteins adopt multi-pass α-helical transmembrane bundle topologies (Figure 6A). Protein interaction network modeling positioned GbZIP16, GbZIP24, and GbZIP36 as central hub nodes interacting with secondary nodes including GbZIP13, GbZIP18, GbZIP26, GbZIP28, and GbZIP44 (Figure 6B).

### 3.7. Expression Patterns Across Tissues and Fiber Development

**Figure 7:**
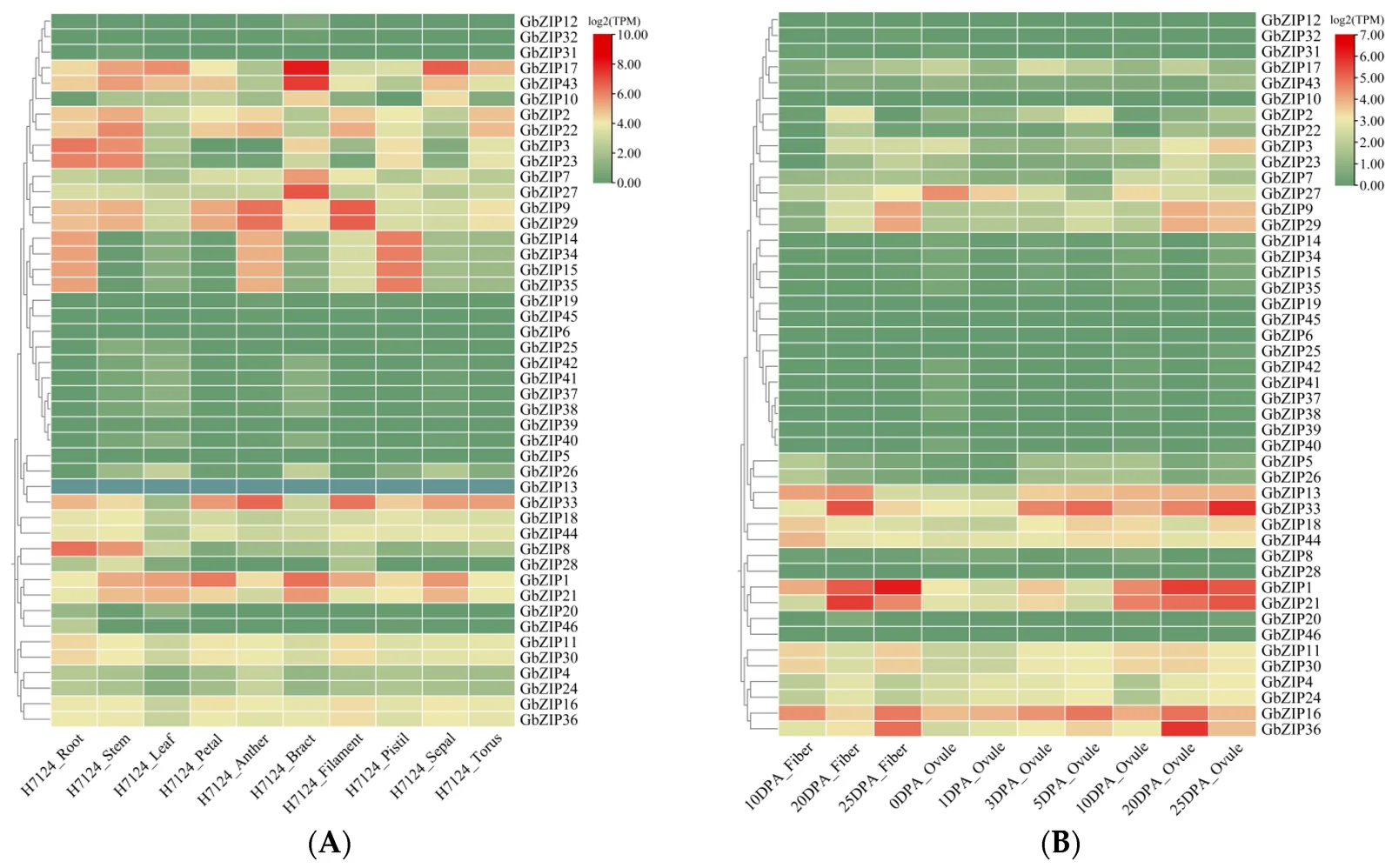
Transcriptional profiling of GbZIP genes. (**A**) Expression across 10 vegetative and floral tissues; (**B**) dynamic expression profiles during ovule and fiber developmental stages.

Tissue-specific RNA-seq profiling showed that *GbZIP3* and *GbZIP23* were preferentially expressed in roots and stems, *GbZIP9* and *GbZIP29* in reproductive floral tissues (anthers and filaments), and *GbZIP14*, *GbZIP15*, *GbZIP34*, and *GbZIP35* in pistils (Figure 7A). During fiber and ovule differentiation, *GbZIP1* and *GbZIP21* showed relatively high expression across multiple stages, while *GbZIP27* displayed stage-specific accumulation, suggesting possible roles in fiber development (Figure 7B).

### 3.8. Abiotic Stress Expression Profiling

**Figure 8:**
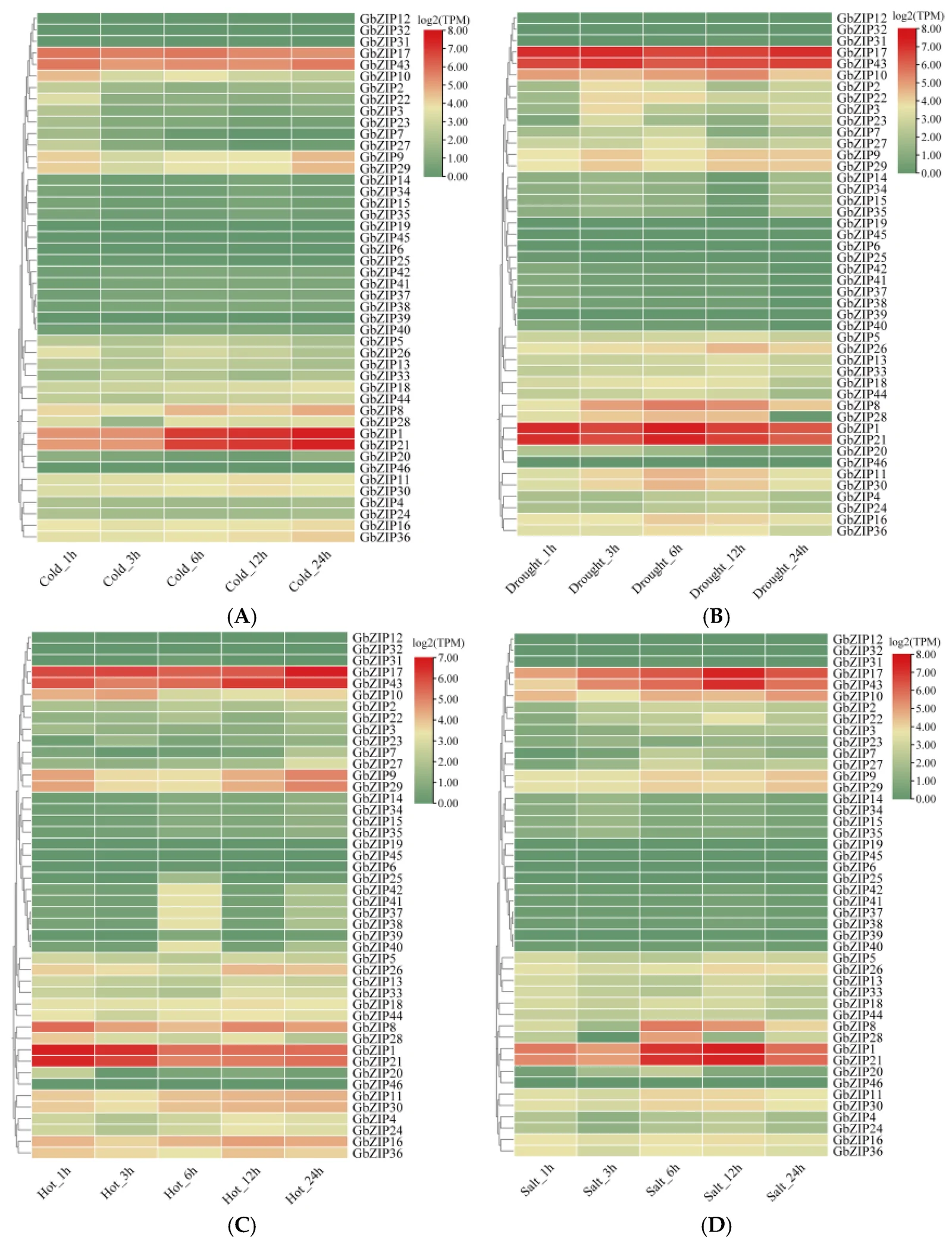
Transcriptional expression heatmaps of GbZIP family members under (**A**) cold, (**B**) drought, (**C**) hot, and (**D**) salt stress treatments.

Under abiotic stress conditions, *GbZIP* members exhibited divergent responses (Figure 8). *GbZIP17*, *GbZIP43*, *GbZIP1*, and *GbZIP21* were strongly upregulated across cold, drought, hot, and salinity treatments. *GbZIP8* and *GbZIP28* responded prominently during late-stage drought, while *GbZIP16* and *GbZIP36* were specifically induced by high temperature and salt stress (Figure 8).

### 3.9. Expression Validation Under Cadmium, V. dahliae, and Combined Stress

**Figure 9:**
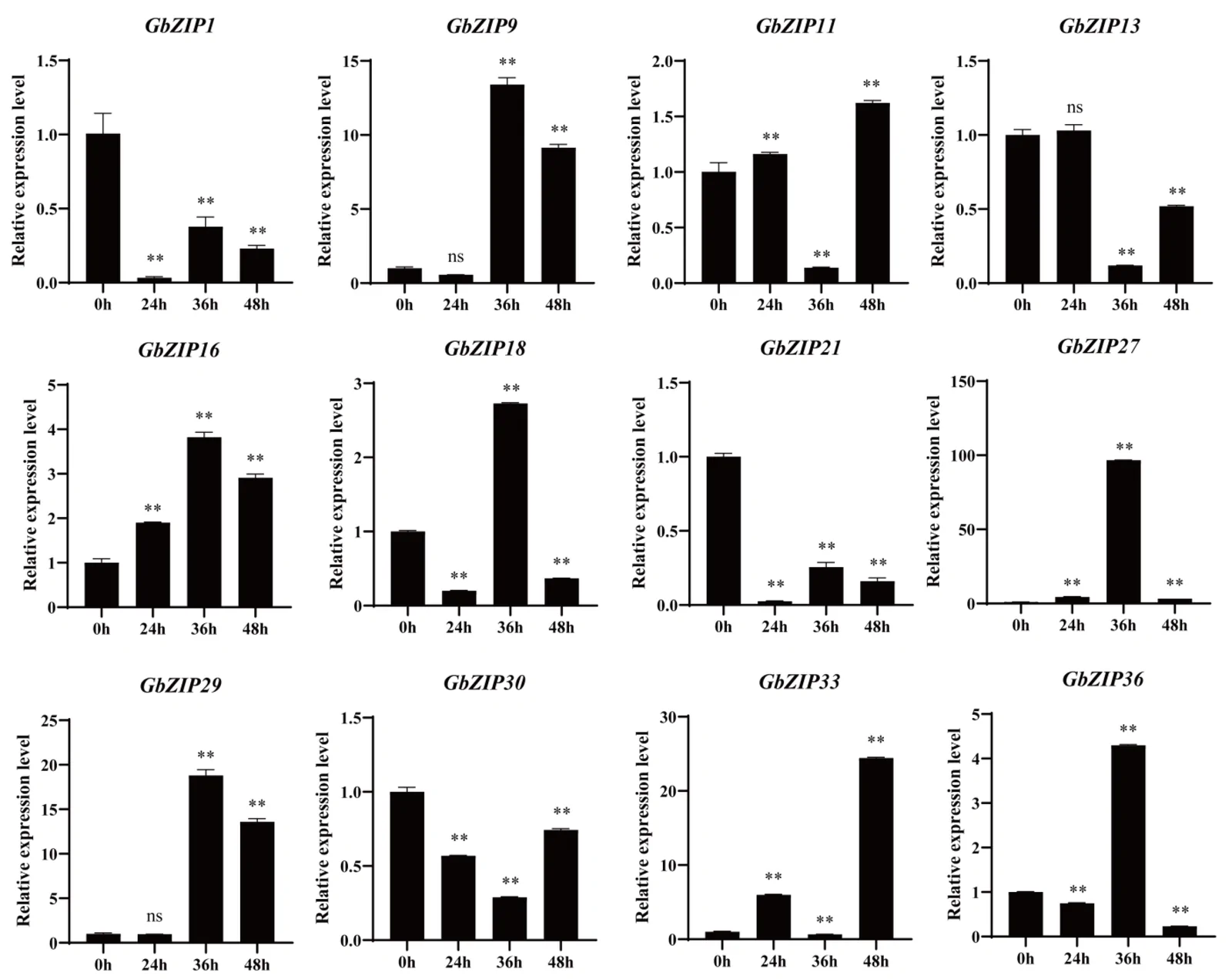
qRT-PCR expression analysis of candidate *GbZIP* genes in roots of *G. barbadense* H7124 at 0, 24, 36, and 48 h under *V. dahliae* inoculation. Relative expression levels were calculated using the 2^−ΔΔCt^ method and normalized to the 0 h control. Error bars denote SD (*n* = 3). Asterisks indicate significant differences compared to 0 h (** *p* < 0.01; *ns*, not significant).

Furthermore, the expression levels of *GbZIP*s under single and combined Cd and *V. dahliae* treatments was analyzed by qRT-PCR. Under *V. dahliae* challenge, *GbZIP9*, *GbZIP16*, *GbZIP18*, *GbZIP27*, *GbZIP29*, and *GbZIP36* were dramatically upregulated at 36 h, with *GbZIP27* displaying a nearly 100-fold induction relative to the control (Figure 9). *GbZIP33* peaked at 48 h with a 24-fold increase.

**Figure 10:**
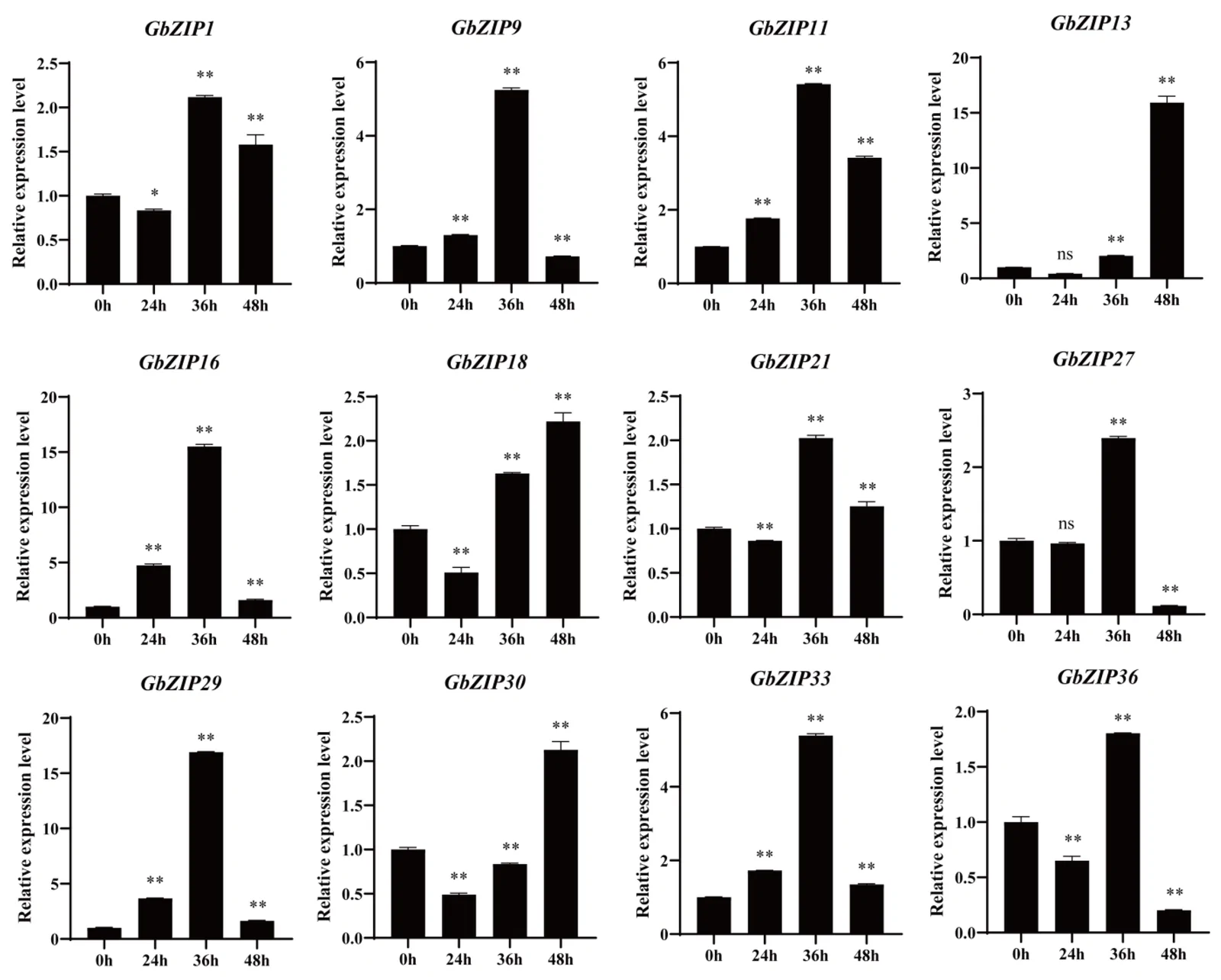
qRT-PCR expression analysis of candidate *GbZIP* genes in roots of *G. barbadense* H7124 at 0, 24, 36, and 48 h under cadmium stress. Error bars denote SD (*n* = 3). Relative expression levels were calculated using the 2^−ΔΔCt^ method and normalized to the 0 h control. Asterisks indicate significant differences compared to 0 h (* *p* < 0.05, ** *p* < 0.01; *ns*, not significant).

Under Cd toxicity, *GbZIP16* and *GbZIP29* reached peak expression at 36 h (15-fold and 17-fold induction, respectively), whereas *GbZIP13* exhibited strong late-stage upregulation at 48 h (16-fold induction) (Figure 10).

**Figure 11:**
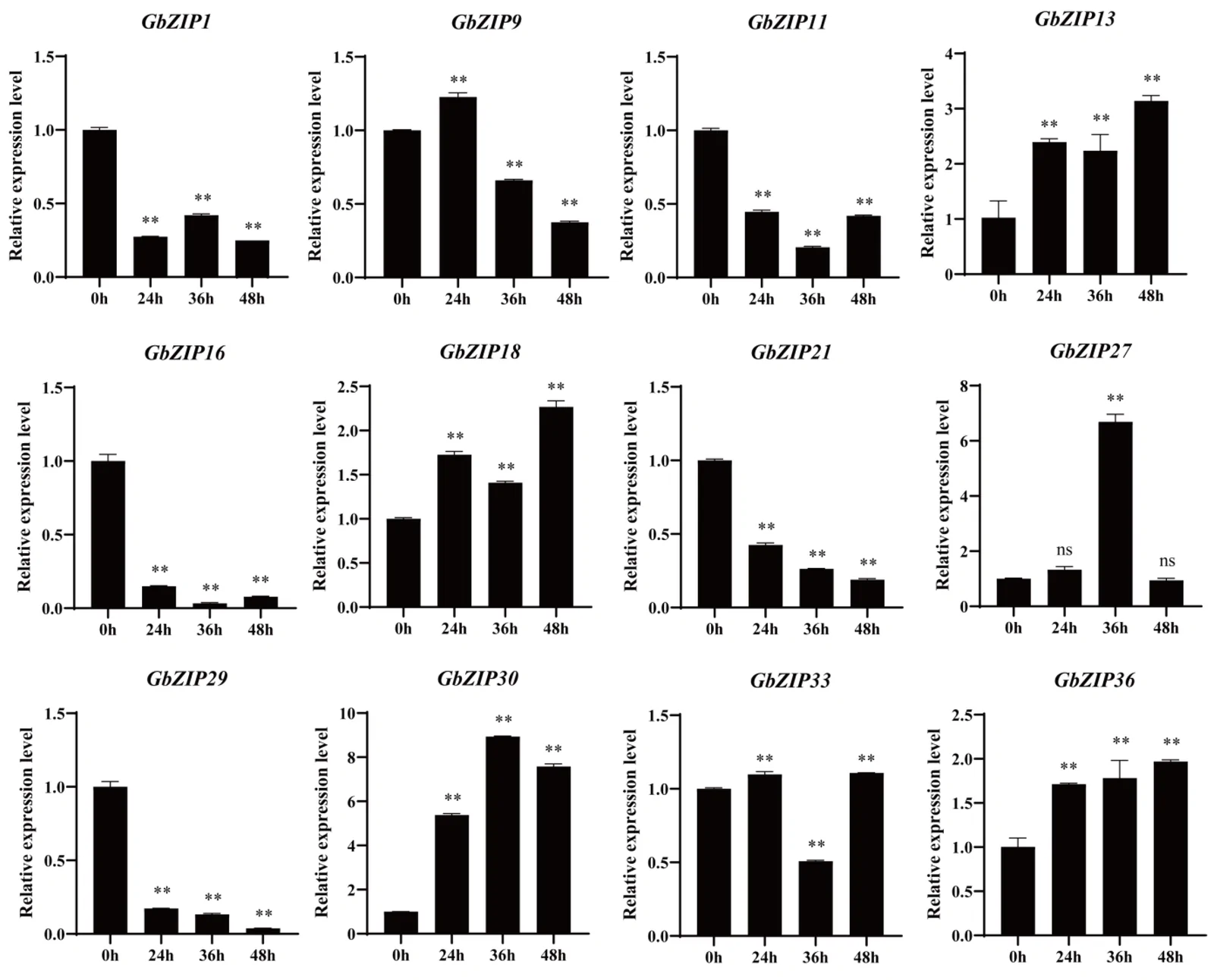
qRT-PCR expression analysis of candidate *GbZIP* genes in roots of *G. barbadense* H7124 at 0, 24, 36, and 48 h under combined Cd-*V. dahliae* stress. Error bars denote SD (*n* = 3). Relative expression levels were calculated using the 2^−ΔΔCt^ method and normalized to the 0 h control. Asterisks indicate significant differences compared to 0 h (** *p* < 0.01; *ns*, not significant).

Under combined Cd-*V. dahliae* stress, *GbZIP13*, *GbZIP18*, *GbZIP27*, *GbZIP30*, and *GbZIP36* showed sustained upregulation, with *GbZIP27* and *GbZIP30* displaying 6.7-fold and 9.0-fold increases at 36 h (Figure 11). Conversely, *GbZIP16* and *GbZIP29*, which were strongly induced under individual stresses, were markedly repressed under combined stress conditions, revealing complex transcriptomic cross-talk (Figure 11).

## 4. Discussion

ZIP transporters act as pivotal regulators of cellular metal ion transport, governing the absorption, intracellular allocation, and systemic movement of essential divalent cations (Zn^2+^, Fe^2+^, Mn^2+^) and heavy metal pollutants such as Cd^2+^ [31]. While ZIP family characteristics have been established in various crops, systematic evaluation in *G. barbadense* has remained incomplete. Here, 46 GbZIP genes were identified and evaluated. Genomic duplication analyses demonstrated that whole-genome/segmental duplications, accompanied by selective tandem duplications, were the primary drivers of family expansion. Structural analysis confirmed high conservation of exon-intron patterns and protein motifs within individual clades, supporting evolutionary functional stability [32].

Promoter analysis revealed abundant stress- and hormone-responsive cis-acting elements across *GbZIP* upstream regions, aligning with transcriptomic findings that *GbZIP* genes respond dynamically to environmental perturbations. Tissue and developmental stage expression profiling revealed specialized physiological roles. High expression of *GbZIP3* and *GbZIP23* in root tissues indicates potential involvement in primary metal uptake from soil, whereas elevated levels of *GbZIP1*, *GbZIP21*, *GbZIP16*, and *GbZIP36* during fiber development suggest that may be involved in fiber elongation and cell wall synthesis [32].

Under abiotic stresses (cold, drought, hot, and salt), multiple *GbZIP* members displayed pronounced induction. Because divalent metal cations modulate reactive oxygen species (ROS) balance, membrane integrity, and stress signaling, ZIP transporters represent potential candidate genes involved in metal homeostasis and stress tolerance [33]. Protein interaction network modeling highlighted *GbZIP16*, *GbZIP24*, and *GbZIP36* as central hubs, suggesting integrated roles in transport and stress regulation.

qRT-PCR validation under heavy metal and pathogen stress further clarified functional specialization. Under Cd stress, *GbZIP16*, *GbZIP29*, and *GbZIP13* exhibited strong time-dependent induction, indicating roles in metal sensing and translocation. In response to *V. dahliae* infection, *GbZIP27* displayed rapid and pronounced upregulation (nearly 100-fold), supporting the connection between metal homeostasis and plant immune responses [34]. Under combined stress of Cd and *V. dahliae* stress, distinct regulatory cross-talk was observed: *GbZIP13*, *GbZIP18*, *GbZIP27*, *GbZIP30*, and *GbZIP36* maintained multi-stress responsiveness, whereas *GbZIP16* and *GbZIP29* were suppressed relative to single-stress treatments. These findings demonstrate that combined biotic and abiotic stresses trigger specific transcriptomic re-programming rather than simple additive responses [35].

## 5. Conclusions

This study provides a comprehensive genome-wide analysis of the ZIP gene family in *G. barbadense*. The 46 identified GbZIP members evolved primarily through duplication-driven expansion while retaining core structural conservation. Expression profiling established their involvement in organ development, fiber maturation, and abiotic stress responses. Furthermore, qRT-PCR analysis further identified *GbZIP13*, *GbZIP18*, *GbZIP27*, and *GbZIP36* as candidate genes responsive to both cadmium stress and fungal infection. In addition, *GbZIP16*, *GbZIP29*, and *GbZIP30* exhibited distinct stress-dependent expression patterns, with *GbZIP30* showing different regulatory behaviors under different stress conditions. Unlike previous studies that only focused on a single stress or a single expression profile, this research incorporates heavy metal stress, pathogen stress, and combined stress into the same analytical framework, which is conducive to revealing the synergistic regulatory patterns of the GbZIP gene in complex environments.

## Figures and Tables

**Table 1:** Physicochemical characteristics and predicted subcellular localization of the GbZIP protein family in *G. barbadense*.

Gene ID	Gene Name	AA	MW (Da)	pI	Instability Index	Aliphatic Index	GRAVY	Subcellular Localization
Gbar_A01G008050.1	GbZIP1	350	37,663.78	5.81	31.09	102.29	0.509	Cell membrane
Gbar_A01G009170.1	GbZIP2	356	38,137.58	6.55	41.12	113.2	0.549	Cell membrane
Gbar_A01G013670.1	GbZIP3	353	37,992.99	8.22	31.14	110.23	0.571	Cell membrane
Gbar_A02G000100.5	GbZIP4	595	62,054.08	7.16	34.31	112.49	0.790	Cell membrane
Gbar_A03G003470.1	GbZIP5	323	34,287.51	7.64	32.51	108.73	0.664	Chloroplast
Gbar_A03G012150.1	GbZIP6	355	38,429.54	8.04	32.38	110.99	0.588	Cell membrane
Gbar_A05G003850.2	GbZIP7	422	45,171.78	5.85	43.66	94.81	0.292	Chloroplast
Gbar_A05G009050.1	GbZIP8	340	36,989.2	6.18	22.85	109.29	0.588	Cell membrane
Gbar_A06G009360.1	GbZIP9	426	45,442.28	6.05	40.18	100.56	0.325	Chloroplast
Gbar_A07G008820.1	GbZIP10	366	39,305.75	6.82	33.16	103.96	0.444	Cell membrane
Gbar_A08G001730.1	GbZIP11	276	29,449.96	9.25	37.91	113.12	0.672	Cell membrane
Gbar_A08G005620.1	GbZIP12	346	36,950.35	6.3	27.99	113.61	0.544	Cell membrane
Gbar_A08G021680.1	GbZIP13	325	34,601.82	7.18	25.62	111.35	0.692	Chloroplast
Gbar_A10G004990.1	GbZIP14	355	38,141.34	8.63	36.18	112.37	0.643	Cell membrane
Gbar_A10G005000.2	GbZIP15	357	38,255.7	8.97	33.31	114.68	0.723	Cell membrane
Gbar_A10G016380.7	GbZIP16	595	62,062.07	6.49	30.54	112.32	0.777	Cell membrane
Gbar_A11G016140.1	GbZIP17	356	38,114.5	6.19	36.1	111.29	0.453	Cell membrane
Gbar_A12G016610.3	GbZIP18	505	54,111	6.29	27.59	86.34	−0.087	Cell membrane
Gbar_A13G000570.1	GbZIP19	351	38,255.32	6.82	31.19	109.17	0.556	Cell membrane
Gbar_A13G014710.1	GbZIP20	330	35,679.53	5.88	32.07	106.94	0.645	Cell membrane
Gbar_D01G008370.1	GbZIP21	351	37,698.68	5.62	27.85	101.71	0.513	Cell membrane
Gbar_D01G009600.1	GbZIP22	356	37,993.42	6.72	40.39	112.67	0.550	Cell membrane
Gbar_D01G014420.2	GbZIP23	372	40,053.41	8.97	30.61	104.35	0.518	Cell membrane
Gbar_D02G000200.1	GbZIP24	595	62,108.04	7.16	34.04	112.96	0.767	Cell membrane
Gbar_D02G014170.1	GbZIP25	355	38,368.41	7.6	30.01	110.68	0.599	Cell membrane
Gbar_D03G014930.1	GbZIP26	323	34,385.61	7.1	33.85	109.91	0.655	Chloroplast
Gbar_D05G004390.1	GbZIP27	422	45,169.85	5.79	44.03	96.42	0.323	Chloroplast
Gbar_D05G009500.1	GbZIP28	239	26,046.67	7.08	26.48	113.1	0.707	Cell membrane
Gbar_D06G009540.1	GbZIP29	426	45,586.5	6.16	41.39	100.8	0.317	Chloroplast
Gbar_D08G001760.1	GbZIP30	276	29,450.9	8.76	38.91	113.12	0.673	Cell membrane
Gbar_D08G005840.1	GbZIP31	221	24,589.19	6.04	34	112.94	0.662	Cell membrane
Gbar_D08G005860.1	GbZIP32	348	37,240.6	6.82	29.68	111.84	0.507	Cell membrane
Gbar_D08G022440.1	GbZIP33	325	34,637.84	7.7	24.2	111.35	0.679	Chloroplast
Gbar_D10G004840.1	GbZIP34	355	38,046.15	8.48	35.99	110.73	0.639	Cell membrane
Gbar_D10G004850.1	GbZIP35	357	38,303.72	8.87	33.01	112.77	0.696	Cell membrane
Gbar_D10G011770.1	GbZIP36	595	62,060.16	6.69	30.51	112.15	0.788	Cell membrane
Gbar_D10G024340.1	GbZIP37	323	34,537.23	8.68	31.85	112.63	0.738	Cell membrane
Gbar_D10G024370.1	GbZIP38	355	38,214.22	8.04	31.03	109.32	0.586	Cell membrane
Gbar_D10G024420.1	GbZIP39	355	38,370.4	7.58	31.05	110.14	0.583	Cell membrane
Gbar_D10G024430.1	GbZIP40	355	38,327.38	8.04	31.16	109.86	0.584	Cell membrane
Gbar_D10G024460.1	GbZIP41	359	38,929.14	8.32	31.34	112.45	0.586	Cell membrane
Gbar_D10G024520.1	GbZIP42	355	38,324.31	7.6	30.51	110.68	0.564	Cell membrane
Gbar_D11G016920.1	GbZIP43	356	38,112.45	5.99	39.15	112.11	0.456	Cell membrane
Gbar_D12G016680.2	GbZIP44	469	50,587.93	6.04	30.69	90.26	−0.092	Cell membrane
Gbar_D13G000360.1	GbZIP45	343	37,316.48	8.17	30.1	115.98	0.640	Cell membrane
Gbar_D13G014440.1	GbZIP46	330	35,743.61	5.75	32.92	107.24	0.658	Cell membrane

## Data Availability

All data supporting the findings of this study are available within the article and its Supplementary Materials. Further inquiries should be addressed to the corresponding author.

## Supplementary Materials

The following supporting information can be downloaded at: https://www.mdpi.com/article/10.3390/biology15171455/s1, Table S1: List of gene-specific primers used for qRT-PCR.

## Abbreviations

The following abbreviations are used in this manuscript:

ZIP	Zinc/iron-regulated transporter-like proteins (ZRT/IRT-like protein)
Cd	Cadmium
qRT-PCR	Quantitative real-time PCR
HMM	Hidden Markov model
CDD	Conserved Domain Database
pI	Isoelectric point
MW	Molecular weight
GRAVY	Grand average of hydropathicity
NJ	Neighbor-Joining
ML	Maximum likelihood
WGD	Whole-genome duplication
PPI	Protein-protein interaction
TPM	Transcripts per million
ROS	Reactive oxygen species
